# Effects of in-Scanner Bilateral Frontal tDCS on Functional Connectivity of the Working Memory Network in Older Adults

**DOI:** 10.3389/fnagi.2019.00051

**Published:** 2019-03-15

**Authors:** Nicole R. Nissim, Andrew O’Shea, Aprinda Indahlastari, Rachel Telles, Lindsey Richards, Eric Porges, Ronald Cohen, Adam J. Woods

**Affiliations:** ^1^Center for Cognitive Aging and Memory, Department of Clinical and Health Psychology, McKnight Brain Institute, University of Florida, Gainesville, FL, United States; ^2^Department of Neuroscience, University of Florida, Gainesville, FL, United States

**Keywords:** tDCS, fMRI—functional magnetic resonance imaging, cognitive aging, DLPFC (dorsolateral prefrontal cortex), working memory, transcranial direct cortical stimulation (tDCS), functional connectivity

## Abstract

Working memory is an executive memory process essential for everyday decision-making and problem solving that declines with advanced age. Transcranial direct current stimulation (tDCS) is a non-invasive form of brain stimulation that has demonstrated potential for improving working memory performance in older adults. However, the neural mechanisms underlying effects of tDCS on working memory are not well understood. This mechanistic study investigated the acute and after-effects of bilateral frontal (F3/F4) tDCS at 2 mA for 12-min on functional connectivity of the working memory network in older adults. We hypothesized active tDCS over sham would increase frontal connectivity during working memory performance. The study used a double-blind within-subject 2 session crossover design. Participants performed an functional magnetic resonance imaging (fMRI) N-Back working memory task before, during, and after active or sham stimulation. Functional connectivity of the working memory network was assessed within and between stimulation conditions (FDR < 0.05). Active tDCS produced a significant increase in functional connectivity between left ventrolateral prefrontal cortex (VLPFC) and left dorsolateral PFC (DLPFC) during stimulation, but not after stimulation. Connectivity did not significantly increase with sham stimulation. In addition, our data demonstrated both state-dependent and time-dependent effects of tDCS working memory network connectivity in older adults. tDCS during working memory performance produces a selective change in functional connectivity of the working memory network in older adults. These data provide important mechanistic insight into the effects of tDCS on brain connectivity in older adults, as well as key methodological considerations for tDCS-working memory studies.

## Introduction

Working memory enables us to remember, manipulate, and reorganize information for short periods of time (Salthouse et al., 1989; Baddeley, 1992, 2003). This executive memory process is fundamental for everyday life and is known to decline with advanced age (Li et al., 2001; Gazzaley et al., 2007). Age-related decline in working memory significantly contributes to loss of independence and decreased quality of life (Mograbi et al., 2014). However, there is currently a paucity of effective interventions for remediating working memory decline in older adults. Transcranial direct current stimulation (tDCS) demonstrates promise as a potential intervention in this domain, but knowledge of the mechanisms underlying tDCS effects on working memory function in older adults remains unexplored.

Non-human primate studies have indicated the importance of the frontal lobes, particularly the dorsolateral prefrontal cortex (DLPFC), in working memory function (Goldman-Rakic, 1987, 1996; Wang et al., 2011). In humans, structural and functional neuroimaging studies support the integral importance of frontal structures in working memory (Boisgueheneuc et al., 2006; Barbey et al., 2013). Reduced structural surface area of right frontal lobe regions in older adults is associated with lower working memory performance (Nissim et al., 2017). In addition, low performing older adults demonstrate significantly increased right lateralized blood oxygenation level dependent (BOLD) response compared to high performing older adults who demonstrate bilateral recruitment of frontal structures (i.e., neural compensation hypothesis; Cabeza et al., 2002). Moreover, functional connectivity of the working memory network decreases with older age (Hampson et al., 2006; Andrews-Hanna et al., 2007; Hampson et al., 2010; Keller et al., 2015). While there is growing evidence supporting the role of frontal structural and functional decline in age-related working memory performance, there is a lack of effective interventions aimed at treating this decline. Such interventions will be necessary for addressing public health concerns related to the increasing age of the world population and the increased prevalence of age-related cognitive decline in the population at large.

Non-invasive brain stimulation techniques have the potential to impact cognitive processes and modify behavior by inducing changes in brain function (Gomes-Osman et al., 2018). tDCS is a safe and painless form of non-invasive brain stimulation that modulates the neuroplastic response of brain tissue and can impact behavior both during and after periods of stimulation through sub-threshold alteration of resting membrane potentials (Nitsche and Paulus, 2000; Monte-Silva et al., 2013; Pelletier and Cicchetti, 2015; Bikson et al., 2016; Woods et al., 2016; Bikson et al., 2018; Knotkova et al., 2019). Prior studies have demonstrated that tDCS can impact and enhance working memory in older adults (Jones et al., 2015; Woods et al., 2019a). For example, Stephens and Berryhill (2016) demonstrated that older adults who received active tDCS over sham (anode-F4, cathode-contralateral cheek) during a working memory training protocol experienced greater benefits on untrained tasks at post-training assessment (Stephens and Berryhill, 2016). Effects of tDCS combined with working memory training have shown to extend and increase training gains (Richmond et al., 2011, 2014; Jones et al., 2015; Stephens and Berryhill, 2016). Recent research also suggests that the efficacy and transfer of tDCS paired with working memory training may be state dependent, with greater transfer occurring when training tasks are more difficult (Gill et al., 2014). These studies demonstrate the promise of tDCS as a potential intervention for age-related working memory decline.

However, there is a gap in knowledge on the mechanism(s) of action of tDCS-based enhancement of working memory in the brain. No study to date has directly investigated the impact of tDCS on functional connectivity of the working memory network. Better understanding of the mechanism(s) of action for tDCS-based working memory enhancement is essential to harness and optimize tDCS as a treatment approach for cognitive decline in older adults. To address this gap in knowledge, we performed a mechanistic study designed to evaluate the acute and after-effects of tDCS on functional connectivity of working memory networks in older adults during N-Back performance (2- vs. 0-back). This study specifically aimed to query the direct impact of tDCS vs. sham on the working memory network, taking steps to stabilize test-retest behavioral and network change across N-back sessions and between conditions (active vs. sham) to focus our inquiry on direct mechanistic effects of an acute tDCS dose on the network. We hypothesized that active stimulation vs. sham would result in increased frontal connectivity during working memory performance in older adults. In addition, we hypothesized that changes in functional connectivity from active tDCS would be greater in the high-effort 2-Back vs. 0-Back conditions.

## Materials and Methods

**Figure 1:**
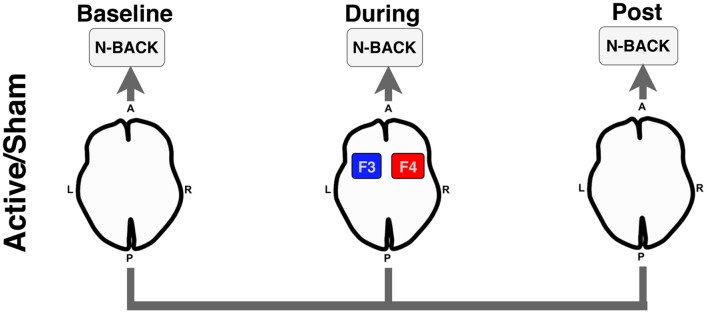
Experimental design for the functional magnetic resonance imaging (fMRI) N-Back task. Participants underwent an N-Back task before (Baseline), during active or sham stimulation (During), and after stimulation was stopped (Post). Anode = red; Cathode = blue. A, anterior; P, posterior; L, left; R, right.

We conducted a randomized double-blinded crossover within-subject study examining the acute effects of bilateral frontal tDCS (2 mA for 12 min; F3/F4) during an functional magnetic resonance imaging (fMRI) BOLD scan. For each stimulation condition (active vs. sham), participants performed three runs (baseline-active/sham, during-active/sham, post-active/sham) of an N-Back working memory task. Participants received each stimulation condition (active and sham) at two separate visits (randomized and counterbalanced), and thus, served as their own controls. Figure 1 illustrates the overall experimental design used in this study.

### Participants

Sixteen older adults between the ages of 61–82 years old participated in this study [n female = 6; mean age (SD) = 71.75 (7.29); mean education = 16.8 (1.92)]. All participants completed a medical history questionnaire and cognitive assessments to meet study eligibility criteria. The Montreal Cognitive Assessment (MoCA) was used to screen for significant global cognitive deficits. Participants were enrolled in the study if they were MRI-compatible, able to receive electrical stimulation on the head, scored at least 20 or more on the MoCA (mean = 26.56 SD = 2.94), were right-hand dominant, and not taking certain medications potentially blocking tDCS excitability effects (i.e., GABAergic, glutamatergic, or sodium channel blockers; McLaren et al., 2018). Participants with a history of neurological disorders, seizures, psychiatric disorders, in active treatment for cancer, previous traumatic brain injury with loss of consciousness greater than 20 min, or any neurodegenerative disease were excluded from the study. The washout period between stimulation visits for each participant was 8.5 days on average (range = 6–16 days). The study protocol was in accordance with the Declaration of Helsinki and approved by University of Florida’s Institutional Review Board. Participants provided written informed consent prior to any study procedures.

### Structural MRI Acquisition

T1-weighted MPRAGE structural MRI scans were obtained prior to BOLD fMRI using a 32-channel head coil (3T Philips MRI). Scan parameters included: repetition time (TR) = 7.0 ms; echo time (TE = 3.2 ms); flip angle = 8°; field of view (FOV) = 240 × 240 × 170 mm; voxel size = 1 mm^3^.

### fMRI Data Acquisition

fMRI scans were acquired on a 3 Tesla Philips MRI scanner using a 32-channel head coil. Task-based fMRI data were collected using a single-shot EPI sequence (36 slices, no gap, TR = 2,000 ms, TE = 30 ms, flip angle = 80°, FOV = 224 × 224 × 126, voxel size = 3.5 mm^3^). Task stimuli were presented on a screen and reflected onto a mirror visible to participants. Participants were trained on the task prior to the MRI session.

### tDCS Parameters and Application

Bilateral frontal tDCS was delivered inside the scanner at 2 mA for 12 min (30 s ramp up/down) during the active condition using an MRI-compatible tDCS device (neuroConn DC-Stimulator MR). The sham condition was identical except that the stimulation period lasted only 30 s. Prior studies that examined 1 mA intensity consistently report increased excitation under the anode and decreased excitation under the cathode electrode in the context of motor-evoked potential TMS paradigms in the motor cortex (Nitsche and Paulus, 2000; Nitsche et al., 2003a,b, 2004). In contrast, increasing the intensity to 2 mA has been shown to produce net excitation under both anode and cathode electrodes (Batsikadze et al., 2013; Reato et al., 2019). Our montage (F3/F4) and parameter selection of 2 mA was chosen to broadly target bilateral frontal brain regions involved in working memory with the intent of producing net excitation under the anode and cathode during active stimulation. Head measurements were performed using the 10–20 International EEG System to identify F3 (cathode) and F4 (anode) locations over left and right DLPFC, respectively. A 5 mm thick electrical conductive paste (Ten20 paste) was applied directly on two 5 × 7 cm^2^ rubber electrodes prior to placing the electrodes onto the scalp before the scan (Khadka et al., 2019; Woods et al., 2019b). A thin layer of paste was also applied to the scalp at the F3/F4 locations for at least 30 min before the scan to allow paste to saturate the skin/scalp and achieve target impedance levels (<2 kOhms). The electrodes were held in place with a rubber Soterix Easy-Head strap. The device was turned on before the start of the second run of the N-Back to allow ramp time to complete prior to start of the fMRI scan. A blinded six-digit code was entered into the device to initiate the randomized stimulation condition for the session. No adverse events were reported during the study.

### N-Back Task

**Figure 2:**
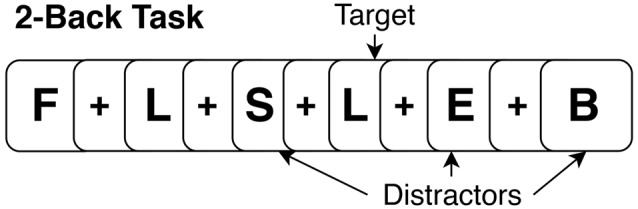
Example of a 2-Back version of N-Back.

During the task period (36 min; 12 min per run), participants performed three runs of the N-Back task (baseline, during, post) for each stimulation condition (active and sham). The task paradigm for each run consisted of four blocks of 2-Back and four blocks of 0-Back presented in a randomized order. In total, 12 blocks of both 2-Back and 0-Back were performed at each visit. During the 2-Back task, participants viewed stimuli of uppercase letters one at a time on the screen with a crosshair (+) as an inter-stimulus interval between stimuli (see Figure 2 for example). The stimulus appeared for 1 s, and the cross hair for 3 s, providing a 4 s window to make a response. Participants were instructed to press a button with their right index finger when the current letter matched the letter that appeared two trials back, and press a different button with their right middle finger when stimuli did not match the 2-Back pattern. The 0-Back was identical to the 2-Back task; however, it lacked the 2-Back pattern component and was used as an attention-control task. For 0-Back, participants were instructed to press a button with their index finger only for the letter “X” and press a different button with their middle finger for any other letter. As this study aimed to mechanistically evaluate the acute impact of tDCS on working memory networks, participants were trained thoroughly on the N-Back task (2- and 0-Back) outside of the scanner to facilitate stabilization of test-retest reliability and comprehension of the task. The intent of this procedure was to facilitate stability of the activation pattern in the working memory network across subsequent runs and between MRI sessions, and isolate detected change in connectivity due to influences from tDCS alone, rather than from the combination of tDCS and test-retest learning. The training consisted of separate 0-Back and 2-Back practice sessions with immediate performance feedback. Afterwards, participants performed an identical version of the task as it appeared in scanner without any feedback. The training took approximately 20 min and was performed before the MRI scan at both active and sham visits. Participants were reminded of instructions while inside the scanner prior to starting the task. N-Back accuracy was analyzed as a percent accuracy score on the 2-Back and 0-Back task. Reaction time was first log-transformed to achieve a normal distribution and the average was taken for each task. Performance (accuracy and reaction time) was evaluated using repeated measures analysis of variance (ANOVA) with stimulation condition (active vs. sham) and time (baseline, during, post) as factors in the model.

### Neuroimaging Pre-processing

Spatial and functional pre-processing was performed using the CONN Toolbox v.2017.f^1^ and SPM12^2^ running in MATLAB 2015b (The Mathworks Inc, Natick, MA, USA). Pre-processing steps were used to segment high-resolution T1-weighted anatomical volumes for each participant into gray matter, white matter, and cerebrospinal fluid, and normalize to Montreal Neurological Institute (MNI) space. Functional volumes underwent realignment, slice-timing correction, and normalization to MNI space using the normalized EPI template image in CONN and a spatial Gaussian smoothing kernel of 8 mm. The artifact detection toolbox (ART) was applied to detect any motion artifacts. Motion parameters from the realignment process were evaluated after pre-processing to identify outliers. Any volumes exceeded a threshold of >3 mm (translation) and >1° rotation were removed from the analysis. Participants included in the analysis did not exceed thresholds for movement. BOLD data was filtered at a bandpass of 0.008-Inf Hz to reduce low-frequency drift and noise effects. Noise correction was performed using the anatomical component-based noise correction (aCompCor) method (Behzadi et al., 2007) implemented in the CONN Toolbox and SPM12. This method extracted principal components from white matter and CSF time series, which were then added as confounds in the denoising step within the CONN Toolbox (Behzadi et al., 2007; Whitfield-Gabrieli and Nieto-Castanon, 2012; Demirakca et al., 2016). This step was used to reduce any physiological and subject movement effects from the time series of interest, and enhance sensitivity, specificity, and validity for subsequent functional connectivity analyses (Behzadi et al., 2007; Whitfield-Gabrieli and Nieto-Castanon, 2012; Demirakca et al., 2016; Fallon et al., 2016).

### Seed-to-Target Regions of Interest (ROIs) Analyses

Using the BOLD signal, we implemented the CONN Toolbox to estimate measures of functional connectivity (Whitfield-Gabrieli et al., 2009; Wei et al., 2017). Previously, the Owen et al. (2005) meta-analysis identified significant activated brain regions during the fMRI N-Back task. We selected the 15 seed regions of interest from this article (Owen et al., 2005) *a priori* to represent the working memory network in our analyses. Spherical regions of interest (ROIs) were created using the WFU PickAtlas GUI in SPM12 (Maldjian et al., 2003, 2004) and the MNI-coordinates from Owen et al. (2005; Table 1); each ROI was created using the volume (mm^3^) reported in the meta-analysis. The default setting of 10 mm radius was used if no volume was reported. It should be noted that the Owen et al. (2005) meta-analyses contained overlap between a subset of ROIs. In each case, radius size varied between identified ROIs. While selecting a single representative ROI would reduce the number of multiple comparisons, potentially increasing the number of significant findings in the current study, we chose a conservative approach aimed at maintaining consistency with the Owen et al. (2005) meta-analysis ROIs. In the case of overlap between left frontal pole and one of the two left dorsolateral prefrontal ROIs, the inclusion of these two overlapping ROIs also provides a degree of ability to examine whether tDCS effects within the frontal pole are related to the portion of the frontal pole consistent with DLPFC or the larger extent of the frontal pole ROI. After pre-processing, denoising, and first level analysis (generalized psychophysiological interaction—gPPI; bivariate regression), second level models for the 2-Back task were analyzed for each of the 15 ROIs with combinations of stimulation session (active or sham) and time (baseline, during, post) as conditions in each model. Target regions only included the spherical ROIs to reduce the number of comparisons and focus on nodes within the working memory network. Model contrasts were created within a single stimulation session (e.g., during-active > baseline-active) for each seed to all targets. Contrasts with significant connectivity changes (FDR < 0.05) were further analyzed between active vs. sham sessions via a *post hoc* paired sample *t*-test to quantify the differences between sessions (e.g., during-active > baseline-active vs. during-sham > baseline-sham).* T*-tests were FDR 0.05 corrected for multiple comparisons. The 0-Back was analyzed in an identical manner.

**Table 1:** Regions of interest (ROIs), Montreal Neurological Institute (MNI) coordinates, radius for spherical ROIs.

Region	*x*	*y*	*z*	Radius
LH DLPFC	−37.75	50.19	13.6	6.2
	−46.26	22.71	18.6	14.3
LH frontal pole	−37.75	50.19	13.6	7.5
LH inferior parietal lobule	−37.09	−47.7	45.58	10
LH lateral premotor	−26.32	6.75	53.46	9
	−45.96	3.1	38.47	10
LH ventrolateral PFC	−31.36	21.11	0.58	10
Medial cerebellum	3.12	−69.09	−24.69	3
RH DLPFC	44.53	38.76	24.43	12.5
RH inferior Parietal Lobule	44.97	−45.49	41.73	12.46
RH lateral premotor	31.96	11.01	49.8	15.83
	31.96	11.01	49.8	10
RH medial posterior parietal	12.77	−63.71	55.28	14.8
RH ventrolateral PFC	35.58	23.26	−3.01	10
Supplementary motor area	−0.588	18.57	40.65	10

*LH, left hemisphere; RH, right hemisphere*.

### Analytical Approach

Using the CONN Toolbox, we examined ROI-to-ROI seed-to-target functional connectivity for each seed to all other specified targets in the working memory network via bivariate regression (gPPI) for 2-Back and 0-Back separately. The N-Back blocks in each run were synchronized with the functional data to only capture the task period and not rest or instruction periods. Fisher-transformed bivariate regression coefficients (beta values) between two ROI BOLD time-series were used to identify significant increases or decreases in functional connectivity between the seed-to-target.

## Results

### Functional Connectivity During 2-Back

**Figure 3:**
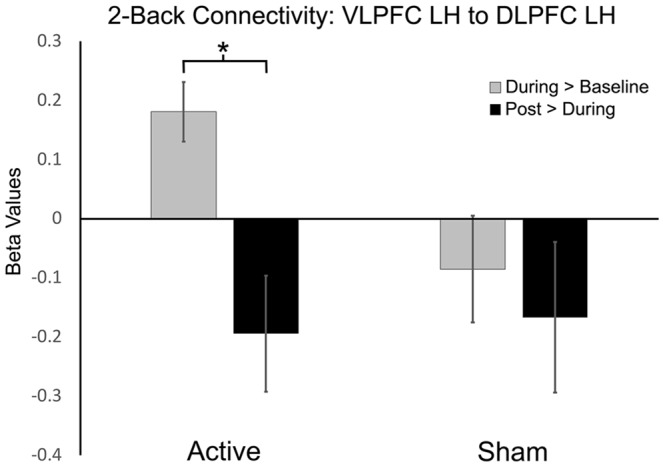
Functional connectivity beta values in active vs. sham session contrasts (seeding left ventrolateral prefrontal cortex, VLPFC to target left dorsolateral PFC, DLPFC). *P-FDR < 0.05.

**Figure 4:**
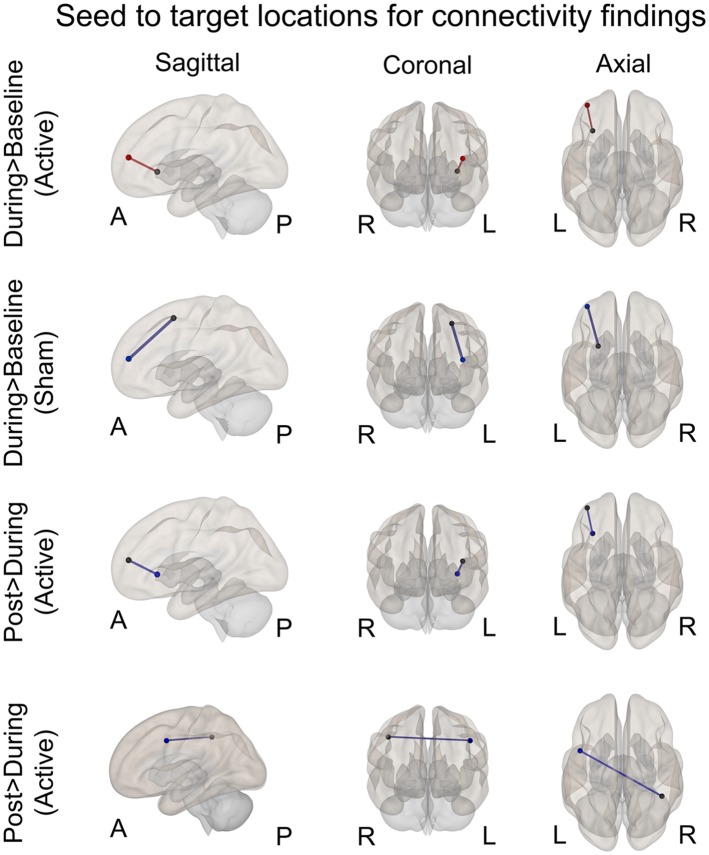
Seed to target locations for significant connectivity findings. See Table 2 for seed to target location names. Nodes colored in black represent the seed location. Nodes color coded in red or blue indicate the target location. Red signifies significantly increased connectivity (FDR < 0.05). Blue signifies significantly decreased connectivity (FDR < 0.05). A, anterior; P, posterior; R, right; L, left.

We examined the effects of active vs. sham stimulation (at two separate visits) across three time points (baseline-active/sham, during-active/sham, and post-active/sham) during the 2-Back working memory task. Seed-to-target analyses demonstrated selective modulation of functional connectivity within the working memory network on the 2-Back task during-active stimulation as shown in Figures 3, 4 and Table 2.

**Table 2:** Significant seed to target connectivity values and test statistics for each contrast.

Contrast	Condition	Seed	Target	Beta	T_(15)_	P-Unc	P-FDR	Outcome
During > Baseline	Active	VLPFC LH	DLPFC LH	0.18	3.59	0.002	0.045*	Increased connectivity
During > Baseline	Sham	Lateral premotor LH	Frontal pole LH	−0.32	−3.4	0.003	0.042*	Decreased connectivity
During > Baseline	Sham	Lateral premotor LH	DLPFC LH	−0.38	−3.28	0.005	0.042*	Decreased connectivity
Post > During	Active	DLPFC LH	VLPFC LH	−0.06	−3.55	0.002	0.049*	Decreased connectivity
Post > During	Active	IPL RH	Lateral premotor LH	−0.17	−3.84	0.001	0.027*	Decreased connectivity

*p-FDR < 0.05.

### During > Baseline

#### Active

A significant increase in functional connectivity was observed during-active stimulation compared to baseline-active when seeding in the left VLPFC targeting left DLPFC (P-FDR = 0.045). On an individual subject level, we observed that 13/16 (81%) participants evidenced increases in connectivity.

#### Sham

We found significantly decreased connectivity during-sham stimulation compared to baseline-sham when seeding in the left lateral premotor targeting the left frontal pole (P-FDR = 0.042) and the left DLPFC (P-FDR = 0.042).

#### Active vs. Sham

Left ventrolateral PFC (VLPFC) to Left DLPFC: *post hoc* analyses comparing during-active > baseline-active to during-sham > baseline-sham demonstrated significantly greater connectivity values in the active condition (*t* = 2.65, *df* = 15, *p* = 0.01, P-FDR = 0.018).

Left Lateral Premotor to Left Frontal Pole: *post hoc* analyses comparing during-active > baseline-active to during-sham > baseline-sham demonstrated significantly lower connectivity values in the sham condition (*t* = 2.77, *df* = 15, *p* = 0.01, P-FDR = 0.047).

Left Lateral Premotor to Left DLPFC: *post hoc* analyses comparing during-active > baseline-active to during-sham > baseline-sham demonstrated significantly lower connectivity values in the sham condition (*t* = 2.68, *df* = 15, *p* = 0.01, P-FDR = 0.047).

### Post > During

#### Active

There was a significant decrease in connectivity between left DLPFC targeting left VLPFC post-active compared to during-active stimulation (P-FDR = 0.049). In addition, connectivity between right inferior parietal lobule (seed) and left lateral premotor (target) was also significantly decreased (P-FDR = 0.027).

#### Sham

No significant changes in connectivity were found when comparing post-sham to during-sham stimulation (P-FDR > 0.05).

#### Active vs. Sham

Left DLPFC to Left VLPFC: *post hoc* analyses comparing post-active > during-active to post-sham > during-sham did not demonstrate a significant difference between sham and active conditions (P-FDR > 0.05).

Right IPL to Left lateral premotor: analyses comparing post-active > during-active to post-sham > during-sham demonstrated that connectivity values were significantly lower in the active condition (*t* = −2.56, *df* = 15, *p* = 0.021, P-FDR = 0.048).

### Post > Baseline

#### Active

No significant changes in connectivity were found when comparing post-active stimulation to baseline-active stimulation (P-FDR > 0.05).

#### Sham

No significant changes in connectivity were found when comparing post-sham stimulation to baseline-sham (P-FDR > 0.05).

### Control Contrast: Baseline-Active vs. Baseline-Sham

No significant changes in connectivity were found between baseline-active vs. baseline-sham stimulation (P-FDR > 0.05).

### Functional Connectivity During 0-Back

The effects of active vs. sham stimulation were evaluated across the three time points during the 0-Back task in an identical manner as 2-Back. Seed-to-target analyses were performed seeding in each ROI targeting all other ROIs in the network. No significant connectivity changes were identified during 0-Back (P-FDR > 0.05). In addition, there were no significant connectivity differences between baseline-active vs. baseline-sham (P-FDR > 0.05).

### N-Back Behavior

Percent accuracy and reaction time on the N-Back task did not significantly differ between active and sham stimulation conditions at the various time points (*p* > 0.05) as reported in Table 3. Evaluation of linear and non-linear fits for performance data demonstrated a trend towards faster reaction time for 2-Back targets (tests of within-subject contrast fit with quadratic equation; *F* = 2.146, *df* = 1.15, partial eta squared = 0.125, *p* = 0.16) and improved 2-Back target accuracy (fit with linear equation; *F* = 3.199, *df* = 1.15, partial eta squared = 0.176, *p* = 0.09) during active-stimulation using repeated measures ANOVA within-subject contrast (time × stimulation condition). Nonetheless, performance was not significantly different for accuracy and reaction time.

**Table 3:** Mean percent task accuracy and standard error on 2-Back and 0-Back for each condition; mean reaction time and standard error in milliseconds for each condition.

N-Back performance (mean, std. error)	Baseline-Active	During-Active	Post-Active	Baseline-Sham	During-Sham	Post-Sham
2-Back accuracy	84% (4%)	81% (5%)	82%(6%)	85% (4%)	85% (3%)	79% (4%)
0-Back accuracy	89% (3%)	92% (4%)	84% (6%)	89% (4%)	89% (3%)	88% (3%)
2-Back reaction time	959.3 (57.2)	847.2 (63.6)	868.5 (39.0)	1015.2 (64.5)	955.5 (62.5)	886.6 (51.4)
0-Back reaction time	791.2 (55.1)	738.5 (60.6)	756.5 (55.5)	830.3 (74.6)	760.3 (62.7)	739.6 (56.9)

## Discussion

To the best of our knowledge, this is the first study to investigate the mechanistic acute and after-effects of bilateral frontal tDCS on functional connectivity of the working memory network in older adults. Our results demonstrate the ability of in-scanner tDCS paired with an N-Back task to acutely modulate functional connectivity in the working memory network. We found increased functional connectivity within the working memory network (seeding left VLPFC targeting left DLPFC) while participants performed the 2-Back task during active stimulation compared to baseline. Moreover, the *post hoc* between-condition analysis demonstrated significantly greater connectivity values in the active condition (P-FDR = 0.018). In contrast, during sham stimulation compared to baseline, we observed a significant decrease in functional connectivity during the 2-Back task (seeding left lateral premotor to left frontal pole and left DLPFC). Importantly, the changes in functional connectivity that occurred during active stimulation only occurred while performing the more challenging 2-Back task, and not during the less challenging 0-Back task. Additionally, increased left frontal connectivity during active stimulation trended towards decreasing post-stimulation, and may suggest that functional connectivity returned to baseline values after stimulation was stopped. This is also potentially supported by a significant decrease in connectivity that was observed at post-active stimulation compared to during active stimulation when seeding left DLPFC targeting left VLPFC. Collectively, these data suggest the ability of acute bilateral F3/F4 tDCS at 2 mA to selectively modulate frontal functional connectivity of working memory related brain regions in older adults during stimulation. These results provide important insight into: (1) selective effects of tDCS on functional connectivity; (2) state-dependent effects of tDCS; (3) the importance of timing tDCS delivery; and (4) effects of 2 mA stimulation under the cathode electrode.

Our results demonstrated an increase in connectivity between two frontal lobe structures (VLPFC and DLPFC) during-active tDCS. Sub-regions of the frontal lobe are essential for manipulation of verbal and spatial knowledge (Barbey et al., 2013). Functionally, the VLPFC has exhibited enhanced activity during various cognitive control processes such as selection, maintenance, and retrieval of goal directed information. The DLPFC has shown critical involvements during task delay periods and facilitates the ability to keep task-relevant information active, in addition to manipulating that information to accomplish the task. Together, the DLPFC and VLPFC demonstrate enhanced activity in cognitive control processes such as monitoring, manipulating, or processing goal directed information, all processes vital to working memory (D’Esposito et al., 1999; Petrides, 2000; Blumenfeld et al., 2013).

Our finding of functional connectivity increase during-active stimulation on 2-Back, and not 0-Back is consistent with previous investigations of state-dependent effects of tDCS. Gill et al. (2014) provided evidence that cognitive enhancement capacity of tDCS may depend on the nature of the task being performed during stimulation (Gill et al., 2014). A challenging and more cognitively demanding 3-Back task produced greater proficiency on a separate working memory task (Paced Auditory Serial Addition Task, PASAT). In contrast, the less cognitively demanding 1-Back task performed during stimulation did not result in an improvement on the PASAT. Our results support prior research that the effects of tDCS are state dependent on more cognitively demanding tasks (2-Back) vs. less demanding tasks (0-Back).

Previous studies examining tDCS paired with cognitive training indicate the importance of timing of stimulation. Martin et al. (2013, 2014) demonstrated that gains from tDCS were significantly greater when stimulation was delivered during an adaptive N-Back cognitive training task vs. stimulation delivered prior to training (Martin et al., 2013, 2014). These data suggest that optimal gains from tDCS may be achieved by “co-stimulating” neural networks through both behavior and electrical stimulation. Our finding of increased functional connectivity during active stimulation, and not at baseline or post stimulation, appears to be consistent with this notion, and further highlights the importance of timing and delivery of tDCS for optimizing brain-based effects. While absence of post-stimulation after effects on functional connectivity do not preclude behavioral effects of tDCS, these data might suggest that there is an optimal window for leveraging brain-based changes of tDCS during the period of stimulation. However, it is important to use caution when interpreting our results in this domain, as our participants only demonstrated trends for improved accuracy and reaction time in the current study.

Previous studies in tDCS and the motor cortex have shown that 1 mA vs. 2 mA of intensity can have differing physiological responses under the anode vs. cathode electrodes by measuring changes in motor evoked potentials (MEPs). At 1 mA, an increase in excitability is typically seen under the anode electrode, whereas the cathode electrode demonstrates a decrease in excitability (Bindman et al., 1964; Nitsche and Paulus, 2000; Nitsche et al., 2003a,b). Batsikadze et al. (2013) demonstrated that 2 mA tDCS can produce a net increase in excitability under both the anode and cathode electrodes (Batsikadze et al., 2013). At present, there are no MEP-like markers within the frontal lobes that indicate physiological change induced via tDCS. However, our finding of increased functional connectivity of left frontal regions during active stimulation may support prior motor cortex findings of increased excitability under the cathode electrode at 2 mA in frontal cortices. If the cathode electrode induced a decrease in excitability in underlying tissue, we might expect to see decreased connectivity, or no change, yet we saw an increase in functional connectivity lateralized to the left hemisphere where the cathode electrode was placed. Therefore, these data appear, at least in part, to support a growing body of research suggesting that 2 mA tDCS may result in increased excitability under the cathode electrode.

When interpreting the results, limitations of the study should be considered. Artifacts in the MRI environment could alter imaging data; we took into consideration how introducing current into the MRI may cause artifacts. Our task paradigm randomized and switched between 2- and 0-Back for during-active stimulation. We did not identify any significant changes in functional connectivity while 0-Back was performed in during-active stimulation. The lack of change in during-active vs. during-sham stimulation on the 0-Back task provides further evidence that the observed connectivity increase was not caused by potential artifacts directly induced by current delivery. This suggests the connectivity changes that occurred during active stimulation on the 2-Back were a result of stimulation. This study did not demonstrate statistically significant behavioral improvements in the active vs. sham session, although a trend for significance and medium effect sizes were present. Our study trained participants prior to in-scanner performance to stabilize the behavioral and network performance across multiple runs, to better enable direct evaluation of acute impact of delivered current flow on functional connectivity in working memory networks. As expected, stable and high levels of performance likely minimized our ability to detect large changes in N-back performance. In addition, limited power due to small sample size likely played a role in these results. It is also important to consider the range of cognitive abilities of the selected sample. The current study used a liberal MoCA cutoff of 20, with an average of 26.5. Thus, the older adult sample in the current study included not only participants that could be classified as “normal healthy” older adults, but also some participants that may not be entirely cognitive normal. Nonetheless, participants were excluded for diagnosis of mild cognitive impairment or dementia. There are also nuances to performing fMRI in older adults that must be considered. The process of aging on the brain may uniquely impact adaptive network mechanisms. Thus, effects found in the current study may not extend to younger adults. Future studies replicated the current findings in younger adults would help to expand our understanding of tDCS mechanisms across the lifespan. As the population studied involves older adults, 25% or more of participants may have beta-amyloid present in the brain. As beta-amyloid can impact functional connectivity, this factor may play a role in our overall results and further stresses the need for follow-up studies in young adults and older adults with corresponding beta-amyloid positron emission tomography.

## Conclusion

The underlying mechanism(s) of action of tDCS and its effects on brain function are not yet fully understood. Our results demonstrated that acute bilateral frontal tDCS selectively modulates functional brain connectivity in the working memory network of older adults. These data indicate the importance of timing of stimulation and the critical influence that state/cognitive effort may have during the period of stimulation. Further research pairing tDCS with unimodal and multimodal neuroimaging is needed to continue elucidating the mechanism(s) of tDCS brain-based effects on cognition.

## Data Availability

The datasets generated for this study are available on request to the corresponding author.

## Author Contributions

NN, AO’S, AI, RT, LR, EP, RC and AW contributed to the manuscript. NN and AW performed data analyses. All authors approved the final version of the manuscript.

## Conflict of Interest Statement

The authors declare that the research was conducted in the absence of any commercial or financial relationships that could be construed as a potential conflict of interest.

## Footnotes

^1^https://www.nitrc.org/projects/conn

^2^https://www.fil.ion.ucl.ac.uk/spm/
